# A Novel Measurement Approach to Experimentally Determine the Thermomechanical Properties of a Gas Foil Bearing Using a Specialized Sensing Foil Made of Inconel Alloy

**DOI:** 10.3390/ma16010145

**Published:** 2022-12-23

**Authors:** Adam Martowicz, Jakub Roemer, Paweł Zdziebko, Grzegorz Żywica, Paweł Bagiński, Artur Andrearczyk

**Affiliations:** 1 Department of Robotics and Mechatronics, AGH University of Science and Technology, al. Mickiewicza 30, 30-059 Krakow, Poland; 2 Institute of Fluid-Flow Machinery, Polish Academy of Sciences, Department of Turbine Dynamics and Diagnostics, Fiszera 14 Street, 80-231 Gdansk, Poland

**Keywords:** gas foil bearing, Inconel alloy, sensing foil, strain field, temperature field, thermomechanical coupling, thermomechanical characterization, rotor dynamics, turbomachinery

## Abstract

Modern approaches dedicated to controlling the operation of gas foil bearings require advanced measurement techniques to comprehensively investigate the bearings’ thermal and thermomechanical properties. Their successful long-term maintenance with constant operational characteristics may be feasible only when the allowed thermal and mechanical regimes are rigorously kept. Hence, an adequate acquisition of experimental readings for the critical physical quantities should be conducted to track the actual condition of the bearing. The above-stated demand has motivated the authors of this present work to perform the thermomechanical characterization of the prototype installation of a gas foil bearing, applying a specialized sensing foil. This so-called top foil is a component of the structural part of the bearing’s supporting layer and composed of a superalloy, Inconel 625. The strain and temperature distributions were identified based on the readings from the strain gauges and integrated thermocouples mounted on the top foil. The measurements’ results were obtained for the experiments that represent the arbitrarily selected operational conditions of the tested bearing. Specifically, the considered measurement scenario relates to the operation at a nominal rotational speed, i.e., during the stable process, as well as to the run-up and run-out stages. The main objectives of the work are: (a) experimental proof for the described functionalities of the designed and manufactured specialized sensing foil that allow for the application of a novel approach to the bearing’s characterization, and (b) qualitative investigation of the relation between the mechanical and thermal properties of the tested bearing, using the measurements conducted with the newly proposed technical solution.

## 1. Introduction

**Figure 1:**
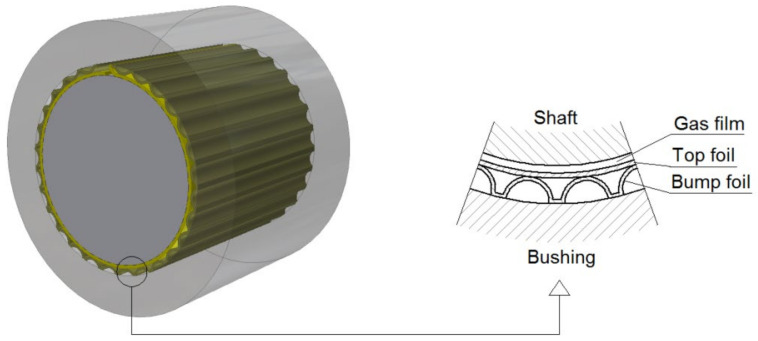
Typical geometry of a bump-type radial GFB. The top and bump foils compose a structural part of the bearing’s supporting layer, whereas the gas film creates a fluidic layer in addition. Both layers ensure the required suspension and elevation capability for the rotating shaft’s journal in a GFB while its operation.

Bearings are one of the critical components of any machine. Very often, they determine the durability and reliability of machinery parts, and their limitations in terms of rotational speed and transmitted loads directly impact the permissible operating range of the entire mechanical system. This is particularly important in modern turbomachines, such as microturbines, whose components are subjected to extreme mechanical and thermal loads. In the case of bearings operating at very high rotational speeds and high temperatures, unique design solutions and materials must be used [1]. Among various types of high-speed fluid film bearings (slide bearings), gas foil bearings (GFBs), also known as air foil bearings (AFBs), play an increasingly important role and exhibit unique properties, which are impossible to obtain with other bearing systems [2,3]. Specifically, GFBs can operate at high temperatures, reaching 600 °C. The high mechanical compliance of the GFBs’ foils makes these bearings able to operate with the shaft misalignment of 0.01 mm at high rotational speeds, i.e., 100,000 rpm. Moreover, the enhanced damping properties of the supporting layer exhibited by the mentioned type of bearings, compared to the classic gas bearings, improve the dynamics of the rotor. Unlike typical slide bearings, where the lubricating medium is primarily oil, GFBs can use almost any gas to operate [3]. They are self-acting (hydrodynamic) bearings, and their geometry changes depending on operating conditions [4]. There are also developed and utilized axial versions of GFBs, known as gas foil thrust bearings [5]. The geometry of a typical radial GFB is shown schematically in Figure 1.

**Figure 2:**
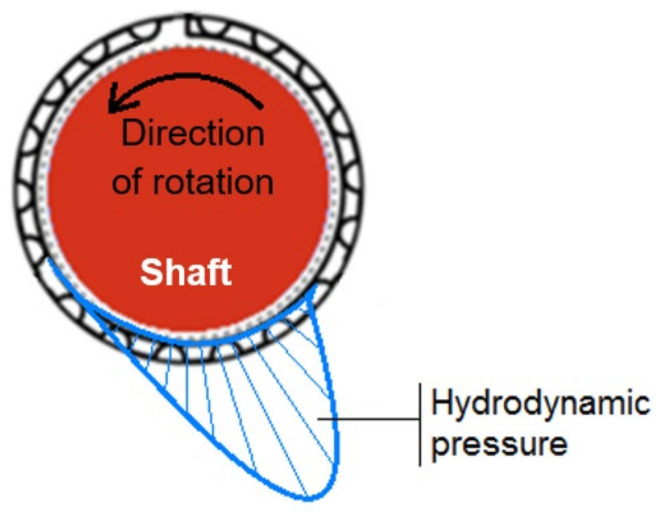
Approximate representation of the hydrodynamic pressure in a GFB.

The most characteristic component of a GFB is a group of thin foils that form a compliant structure with variable geometry. When the shape of the top foil, supported in many small-sized areas by the bump foil, is adjusted to the current position of the journal, the load is transferred to a large area, and the unit pressures are low. When the bearing is working and the journal rotates at a certain speed, it is lifted by a gaseous lubricating film, which is formed due to the hydrodynamic effect. Thus, above a certain speed (called the “lift-off speed”), the journal is no longer in direct metal-to-metal type contact with the top foil and no wear occurs in the bearing. Figure 2 shows an example of a hydrodynamic pressure profile that develops during the operation of a GFB. For clarity, this profile does not consider local changes in the locations of top foil support regions.

Thanks to their specific design, GFBs can self-adapt to variable operating conditions [3,4,6]. However, it should be mentioned that GFBs are only suitable for lightly loaded and high-speed rotors due to the relatively low load capacity, which results from the low viscosity of the gases [6,7]. Among the various applications of GFBs in oil-free turbomachines, in which rotational speeds can exceed 100,000 r/min (100 krpm), their use in gas/vapor microturbines and turbocompressors is also known [3,8,9,10].

There are many design variations of gas foil bearings, and they can be modified to achieve desirable mechanical properties or a better heat transfer [7,11]. GFBs can therefore differ significantly in the geometry and layout of the bump foil, and other flexible elements composed of different materials or a metal mesh can be used instead [11,12,13,14]. In addition, the modified contact conditions between the bumps and the sleeve and the use of additional springs change the stiffness and damping properties of the whole bearing [15,16]. In order to actively change the properties of foil bearings during their operation, more advanced designs are proposed, where smart materials, such as shape-memory materials, piezoelectric materials, or thermoelectric materials, are used [17,18,19]. Other structural modifications of GFBs have been presented in a number of papers [20,21,22,23,24].

GFBs also have some disadvantages. One of the major drawbacks is their sensitivity to changes in operating temperatures. Changes in foil geometry due to the thermal effects can affect the stable operation of the bearing. Thermal deformations of the top foil and bump foils can also interfere with the formation of a gaseous lubrication film, leading to accelerated wear or even damage of the bearing. These phenomena are very complex and are still required to be studied when thermal, flow, and structural behavior, as well as the interactions between them, occur. Changes in the mechanical characteristics of GFBs, which were caused by temperature shifts, have been confirmed experimentally by many researchers [6,25,26,27] and concerned bearing parameters, such as stiffness, damping, load capacity, and coefficient of friction. The relationships between temperature, rotational speed, assembly preload, lift-off speed, and torque are also discussed in articles [28,29]. In reference to the above-cited works, it is worth mentioning, that the preload affects both the lift-off speed and the thickness of the gaseous film. These parameters, in turn, effectively influence the damping and stiffness of the foil assembly, as well as the friction torque of the bearing. The control of preload can be performed in several ways, e.g., by selecting the nominal installation clearance for the shaft’s journal (within the range from −0.02 mm up to 0.02 mm), with the shape and number of bumps of the bump foils or via selection of the materials used to construct the components undergoing thermally-controlled geometric expansion. However, there is still a lack of publications on thermomechanical couplings observed in GFBs. This results, among other things, form the difficulty of accurately measuring the performance of thin flexible foils when bearings operate. Although such studies can be performed using advanced numerical models, a direct measurement of the entire deformation zone and foil temperature still presents technical difficulties. An overview of GFB modeling methods, which take into account temperature distribution, can be found in papers [2,7,30]. Examples of thrust GFB models that allow the analysis of thermal, flow, and structural phenomena are also presented in papers [31,32].

In the available literature, there are many publications in which the numerical methods used to analyze the displacements, deformations, and stresses of foils are discussed. For this purpose, the finite element method (FEM) is most often used, which is implemented in an in-house developed computer program or commercially available software. In this way, all the operational parameters of the foils, including their displacements, deformations, and stresses, can be determined at any point. Specifically, among other applications, FEM has been successfully employed to investigate the multiphysics properties of GFBs, including thermo-hydrodynamic analysis [28], characterize the bearing’s adaptive and non-linear structural properties [4,6], and conduct study on an active bump-type foil bearing [24], as well as GFB dynamics [33,34].

Authors of experimental studies carried out on foil bearings most often focus their attention on determining more general working conditions and the bearing’s properties or the entire rotating system, such as the bearing’s structural stiffness, shaft position, journal and bushing orbits, gas film thickness, or critical rotor speeds [34,35,36]. The mechanical parameters that determine the foils’ operation are not the subject of such studies, as this would require very advanced measurement techniques—such as, for example, vision methods [37]—or the integration of measuring elements with the examined object [38,39]. Therefore, new solutions and measurement methods are constantly being sought in this field of research.

The great importance of thermally induced phenomena in GFBs and their impact on the mechanical properties motivated the authors of this article to develop a new measurement method that uses a specialized sensing top foil composed of a superalloy, Inconel 625, and, then, perform the thermomechanical characterization of the prototype installation of a gas foil bearing to the extent not yet taken into account. Particularly, extraordinary measuring properties were sought for the GFB’s structural components acting as the hosts for the integrated sensors. As successfully experimentally proven, a standard structural component, i.e., the bearing’s top foil, gained new functionality and, therefore, become a functional component as well. This part enables the acquisition of temperature and strain field readings and characterizes their spatial distribution. The identification of the temporal courses for the above-mentioned critical physical quantities makes it possible to track the actual condition of the bearing.

The temperature measuring methods currently used for GFBs are mainly supported by a thermal imaging camera or several, preferably industrial, thermocouples rarely placed at different locations of the bearing [7,32,40,41]. However, more reliable measurement techniques are needed, which would allow a denser arrangement of sensors. The main objectives of this study are to experimentally validate the proposed measurement approach, which is based on data obtained from thermocouples and strain gauges integrated with the top foil, as well as qualitative investigation of the relation between the mechanical and thermal properties of the tested bearing, making use of the measurements conducted with the newly proposed technical solution.

The temperature and deformation measurements, which were performed using a prototype system developed by the authors, should allow for further studies of the thermomechanical effects occurring in the GFB, including, for example, the identification of new multiphysical relationships. This will make it possible to determine the conditions necessary to maintain long-term stable bearing operation at different rotational speeds and loads.

The paper is composed as follows. After the introductory section (Section 1), Section 2 discusses the novelty of the developed sensing foil, which makes use of the components dedicated to accurate temperature and deformation measurements, and describes a prototype installation of the tested GFB. The presentation of both the measurement system and investigated cases declared within the assumed measurement scenario is addressed in Section 3. The considered scenario relates to all stages of the operating bearing, including: run-up, steady-state run at a nominal rotational speed, and run-out. This is followed by Section 4, which reports the results of the experiments conducted for the operation scenario selected for the examined GFB. The final sections (Section 5 and Section 6) discuss the obtained outcomes and a summary of the research conducted, complemented by the key conclusions.

## 2. Novel Prototypes of GFB and Specialized Sensing Top Foil 

In the following, the novel features of the utilized GFB’s test stand, equipped with the developed sensing top foil composed of a super alloy, Inconel 625, are briefly presented and discussed to highlight application areas available for the proposed measurement approach. As mentioned in the introductory part of the paper, new components, i.e., thermocouples and strain gauges, that were boned to the GFB’s structural part, have advantageously enabled accurate temperature and deformation measurements, and, hence, allow for the assessment of the bearing’s operational conditions in the mechanical and thermal domains, simultaneously.

**Figure 3:**
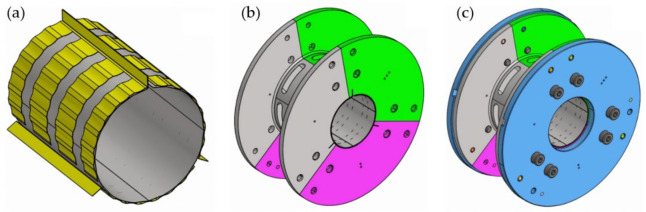
CAD model of the developed prototype a GFB: (**a**) top foil (seen as the inner grey cylindrical component) covered by the set of three bump foils (marked in yellow), (**b**) spool-shaped bushing composed of joined tierces (visualized with three different colors), (**c**) complete assembly equipped with two side flanges (marked in blue).

Figure 3 presents the CAD model of the elaborated prototype installation. As visualized, several changes were introduced into the classical construction of a GFB to allow for demanded extraordinary measurement capabilities in the investigated bearing [1,2,3]. First, the geometry of the bump foils was adapted to provide sufficient space for the sensing components installed on the outer surface of the top foil. It is worth mentioning, that even though the area of the bump foils was significantly reduced, the bearing operated correctly providing carrying load capability. Advantageously, there was not experienced any effect that would significantly disturb both the process of developing the air film during the run-up stage and stable operation of the tested bearing. No sagging effect was indicated as well. The number of the circumferentially distributed supporting areas for the bump foils was not changed compared to the initial GFB’s design; however, the authors are aware of the presence of significant changes in the foils’ stiffness and damping properties induced by the bump foils’ incisions. Moreover, the bushing has undergone considerable remodeling, including such additional machining operations as: removal of a significant part to become a spool-shaped component, and milling 18 holes divided into three tierces. All the above-listed operations eventually allowed for the installation of the sensing top foil as well as for radial guidance of all wiring coming out of the thermocouples and strain gauges. Again, the experimental tests performed to complete the run-in process of the top foil confirmed sufficiently high stiffness of the newly-constructed bushing and correct structural behavior of the developed GFB. Specifically, the expected temporal courses of the measured friction torque and temperature were registered during experiments [42], as shown in Section 4. After the running-in process was completed, the values of the measured quantities expectedly dropped. Following the authors’ previous experience, it should be expected that any significant change of the bearing’s elasto-damping properties registered during the running-in process should lead to the considerable variations of the friction torque and temperature.

**Figure 4:**
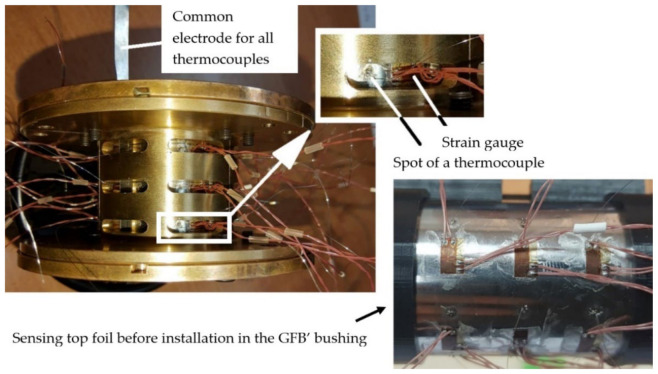
Assembled prototype GFB’s bushing (on the left) and sensing top foil before installation in the bearing (on the right). The integrated thermocouples and strain gauges are visible through the openings in the spool-shaped tierces of the GFB’s bushing that were machined to allow radial guidance of the sensors’ cabling. On the sensing top foil, there were visible spots caused by the gluing process of the strain gauges. However, the excessive amount of glue was removed from the area of contact between the top and bump foils with chemical agents right after completion of gluing process. Effectively, no unexpected behavior of the bearing was indicated due to both the possible change of friction properties and local over-stiffening.

**Figure 5:**
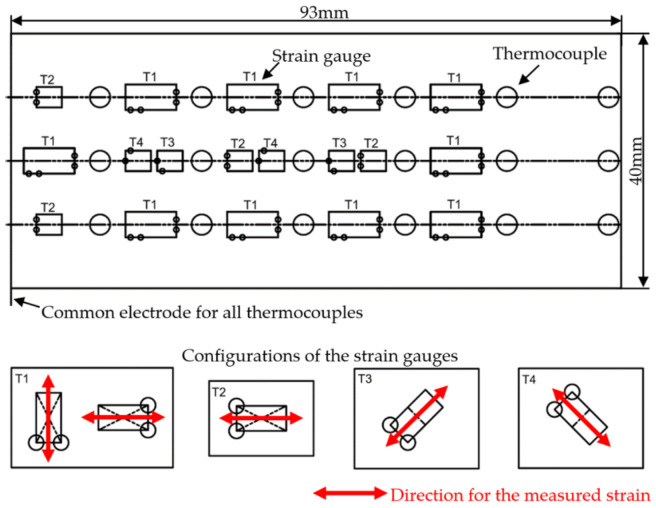
View of the flattened prototype sensing top foil to visualize the circumferential and axial distribution of the integrated thermocouples and strain gauges. Four configurations of the strain gauges were considered to allow multiaxial strain acquisition. However, in the current study, the authors only investigate the temperature and strain change based on the centrally axially located sensors to provide preliminary results and general thermomechanical characterization for the inspected bearing, as announced in Section 3.

Figure 4 presents prototypes of novel constructions of both GFB and the specialized sensing top foil. As previously mentioned, additional circumferentially and axially distributed openings provide access to all sensing nodes and ensure enough space for cabling guidance. The developed specialized top foil makes use of the sensing components installed on its out surface and is dedicated to accurate temperature and strain measurements. The thermocouples are constructed using electrofusion welding with a 0.1 mm-diameter platinum-rhodium alloy. A single piece of Inconel 625 alloy, being a part of the top foil that comes out of the bearing, was used as a common electrode for all thermocouples. Strain gauges, in turn, were glued on the top foil with a dedicated adhesive to provide the necessary mechanical coupling for force and deformation transfer and protection against over-stiffening that would prevent the bearing from normal operation. The geometric pattern used to distribute all the sensing components on the top foil is shown in Figure 5. The main geometric parameters of the tested bearing are collected in Table 1.

**Figure 6:**
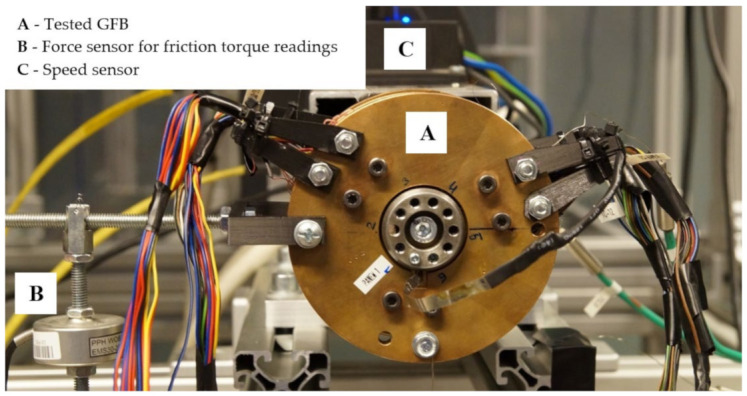
Complete GFB prototype assembly mounted on the electrospindle’s shaft. A simple 125 mm-long lever structure allowed for indirect friction torque measurement via a force sensor. Dedicated 3D-printed clamps, seen at both sides of the bearing, held the sensor cables. The fully assembled and equipped bearing weighed 1165 g. Apart from the gravitational force generated by the bearing itself, no additional load was considered in the test. Hence, the total load for the GFB was estimated to equal 11.43 N, considering the gravitational acceleration 9.81 m/s^2^. The digits handwritten on the bearing close to the shaft approximate the circumferential locations for the temperature sensors.

Figure 6 presents a complete assembly of the constructed GFB prototype mounted on the electrospindle’s shaft. It is worth noting that the inner surface of the top foil was covered with a protective polymer layer AS20, which is based on Teflon with additives. More detailed description of the layer’s properties and application can be found in [42]. Similarly, the shaft’s journal was subjected to a hardening operation, making use of chromium oxide Cr_2_O_3_. The described surface thermal treatment reduced the friction torque and led to a smaller wear of the cooperating structural metallic parts that appeared during the run-up and run-out stages of GFB’s operation.

## 3. Measurement System and Investigated GFB’s Operation Scenario

This section provides the details of the measurement system utilized for temperature, strain, friction torque, and rotational speed readings acquisition, and presents an overview on the bearing’s operation scenario arbitrarily selected by the authors to investigate the GFB’s thermal and mechanical behavior for various operational conditions experienced within a single run of the bearing, including the stages: run-up, steady-state run at a nominal rotational speed, and run-out.

### 3.1. Experimental Test Stand

**Figure 7:**
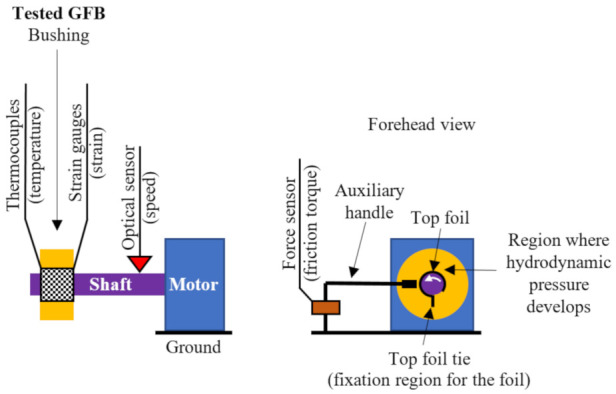
Schematic view of the freely-suspended configuration used for thermal and mechanical characterization of the tested GFB. The specialized sensing top foil—marked with the chessboard pattern—was mounted in the tested GFB and enabled temperature and strain measurements. The white arrow drawn on the shaft indicates its direction of rotation.

**Figure 8:**
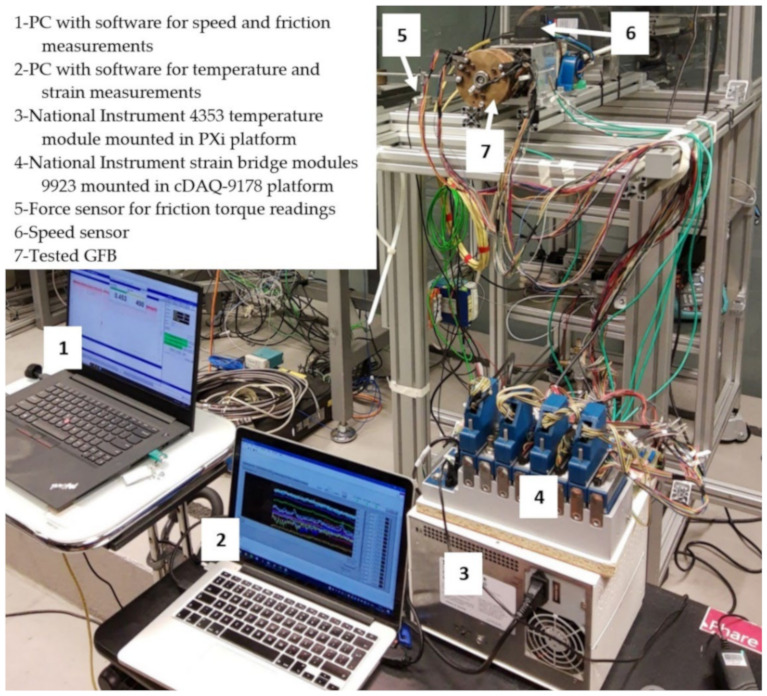
Measurement system used for the acquisition of temperature, strain, friction torque, and rotational speed readings.

**Figure 9:**
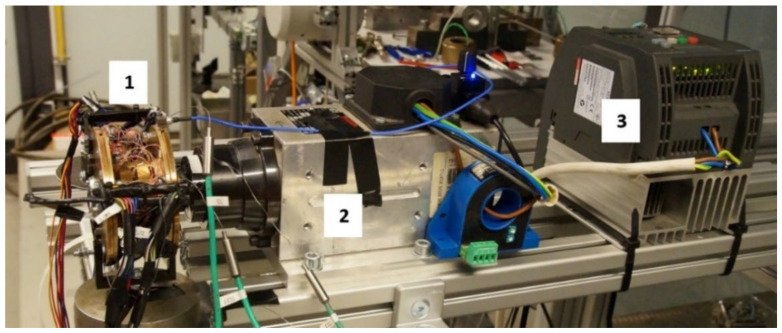
Close-up view of the bearing (**1**), electrospindle (**2**) and inverter (**3**).

A freely-suspended configuration of the tested GFB was considered during measurements, as visualized in Figure 7. A photograph of the prototype installation is presented in Figure 8. A close-up view of the selected components of the test stand, including the GFB, is shown in Figure 9.

This above-mentioned operational configuration, also known as floating configuration, is usually applied for the initial inspection of the bearing’s characteristics. It does not use the housing, nor is another bearing support considered in a test stand. Effectively, the lack of rigid fixation allows a GFB to adjust the orientation and position of its structural parts in the supporting layer and, hence, complete the process of running-in for the top foil safely. To ensure proper operation of the bearing, i.e., the generation of the air film that elevates the GFB over the shaft’s journal, the top foil tie must lie at the bottom.

To conduct the current research on the thermomechanical characterization of GFBs, the authors have extended the measurement capabilities of the previously investigated bearing’s prototype installation, which is described in the work [43]. In the present work, the strain bridge modules were considered for the deformation measurements. Due to the limitations regarding the number of measurement channels available in the utilized hardware, not all strain gauges—among the sensors presented in Figure 5 that cover the entire surface of the top foil—were active to provide strain readings simultaneously. The authors arbitrarily selected the groups of the sensors used in the performed measurement campaigns. Specifically, in the present study, only the strain and temperature sensors lying in the middle column, i.e., centrally axially located in the top foil, were considered to provide preliminary results and general thermomechanical characterization for the inspected bearing. Hence, for the sake of clarity regarding data interpretation, the cross-sectional views for the circumferential distributions of the two above-mentioned quantities identified only within the GFB’s central transversal plane are analyzed.

Characteristics of the temperature and strain sensors used to provide the experimental results in the current study are presented in Table 2. In the case of the thermocouples, regularly distributed sensing nodes were available every 60 deg. along the top foil’s circumference to uniformly cover the angular domain visible in a cross-sectional view. Contrarily, the circumferential distribution of the strain gauges employed for the strain readings was irregular. On one hand, not all sensors were available for the considered measurement scenario due to the limitations resulting from the number of strain bridge modules or identified strain gauges malfunctions. On the other hand, by default, the sensors providing the strain measurement in the axial direction (i.e., the ones of the configuration T1) were eliminated as not providing useful information for the circumferential distribution of the analyzed quantity. Nonetheless, almost the entire angular domain was covered with the strain readings to allow for further interpolation and extrapolation of this quantity along the top foil’s circumference.

**Table 2:** Thermocouples and strain gauges centrally axially localized in the top foil used to collect measurement data for the current study.

Type of Sensor	Angular Position with Respect to the Localization of the Top Foil Tie [deg.]	Orientation of the Strain Component with Respect to Circumference [deg.] (Row along Circumference from the Top Foil Tie/Sensor Configuration)	Comments
Thermocouples	42.5	Not applicable	All thermocouples have a common electrode at the top foil tie
	102.5		
	162.5		
	222.5		
	282.5		
	342.5		
Strain gauges (sequentially denoted in Figure 10: SG1, … SG5)	62.5 82.5 182.5 202.5 267.5	−30 (Row 2/Configuration T4) 30 (Row 2/Configuration T3) 30 (Row 4/Configuration T3) 0 (Row 4/Configuration T2) 0 (Row 5/Configuration T1)	Skipped sensors: Row 1/Configuration T1—angular position 7.5 deg., orientation 90 deg. Row 5/Configuration T1—angular position 247.5 deg., orientation 90 deg. Not available sensors: Row 1/Configuration T1—angular position 27.5 deg., orientation 0 deg. Row 3/Configuration T2—angular position 122.5 deg., orientation 0 deg. Row 3/Configuration T4—angular position 142.5 deg., orientation −30 deg.
		Please see Figure 5 for reference	

**Figure 10:**
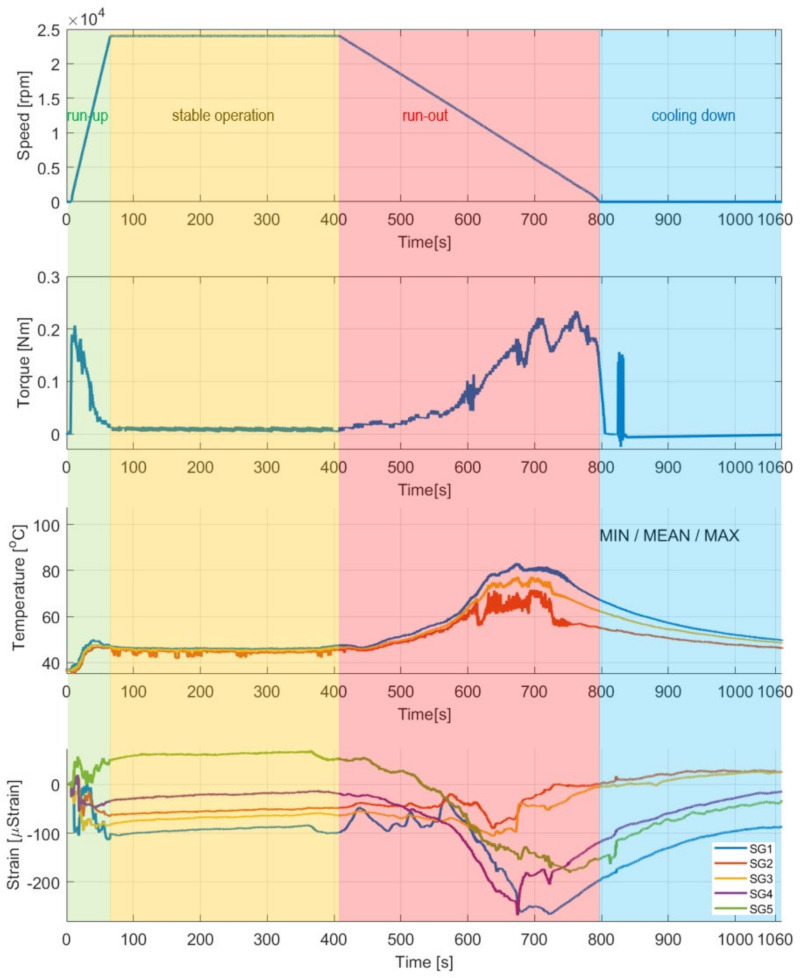
Temporal courses for the investigated operational characteristics of the GFB’s prototype. From the top down to bottom: rotational speed of the shaft’s journal, friction torque, temperature, and strain for the circumferentially distributed measurement points in the sensing top foil. Localization of the strain gauges SG1 up to SG5 is provided in Table 2.

It should be noted that only circumferential components of the measured strains were analyzed by the authors in the current study. They are considered dominant ones in terms of the top foil’s behavior and the capability of developing the air film [44,45]. Specifically, the circumferential curvature of the top foil is much more prone to both the external load and the hydrodynamic effect. In contrast, axial deformations of the top foil require a higher level of mechanical and thermal excitation. Similarly, a general assumption was made by the authors that the circumferential and axial directions for the measured strains coincide with the directions of the principal strains following the specificity of the top foil’s behavior. Finally, initial non-zero strains, registered for all strain gauges before the initialization of the measurement session, were canceled. Hence, the changes in the measured strains were primarily studied in the present research to infer the GFB’s operation.

A precise 24-bit module PXi-NI4353 was utilized for temperature measurement. However, the built-in procedure of automatic compensation, making use of the integrated thermistor that is dedicated to standard industrial thermocouples, could not be applied in the case of the welded sensors. Consequently, the authors identified their properties with a calibration procedure. A constant value of 25 °C, with its experimentally confirmed variation not greater than +/−1 °C, was assumed at the cold-junction, i.e., at the common electrode for all thermocouples. Nonetheless, the estimated maximum error regarding relative temperature measured with the integrated sensors equals 0.5 °C for the temperature range 0 °C up to 100 °C.

### 3.2. Measurement Scenario Selection

The considered measurement scenario (i.e., GFB’s operation scenario) was arbitrarily selected to allow the investigation of all stages of operation of the tested prototype bearing. This scenario assumed that the tested bearing undergo a single 1070-s-long cycle of its complete run, including:

- Measurement process initialization at non-rotating shaft—lasting for 7 s;
- Run-up stage—for 60 s with the rotational acceleration 6.67 Hz/s (6.67 r/s^2^);
- Stage of stable operation at 24,000 r/min (24 krpm, i.e., 400 Hz)—for 340 s;
- Run-out stage—for 390 s with the rotational acceleration –1.03 Hz/s (–1.03 r/s^2^);
- Cooling down at a non-rotating shaft and the process of completing the measurements—for 273 s.

Among all the temperature and strain readings experimentally acquired during the considered GFB’s operation scenario, the authors have intentionally selected some of the measurement results for discussion in the current paper due to their significant contribution to the understanding of the GFB’s thermomechanical behavior. Specifically, the experimental outcomes obtained while changing the bearing’s operational conditions, i.e., during the run-up and run-out stages for the processes of both developing and losing the air film are presented (in Section 4) and then analyzed (in Section 5). Moreover, the stage of the GFB’s stable operation, i.e., its nominal rotational speed, considered to be 24 krpm for the performed tests, is investigated. This speed guaranteed the presence of the air film continuously elevating the bearing over the shaft’s journal as desired for the configuration of a freely-suspended GFB. Finally, the cooling down stage is also addressed to identify a plastic deformation of the top foil. Adequately, several case studies were selected for more comprehensive analysis based on the temporal plots drawn for the experimental readings of the temperature, as discussed in detail in Section 4. As previously experienced by the authors, the temperature may be considered a reliable indicator of the current operational state of the bearing [43].

## 4. Experimental Results

In the current section, the outcomes of the experiments conducted for the considered scenario of the bearing’s operation are reported. Temperature and strain results visualization was performed using both temporal plots (shown in Section 4.1) and transversal cross-sectional views to enable the presentation of circumferential distributions of the above-mentioned quantities being caught at given time moments (presented in Section 4.2). The transversal cross-section located at the center of the top foil was taken into account to characterize the GFB and formulate adequate conclusions in Section 5.

Specifically, the time moments at which the air film developed and was lost are of the authors’ particular concern. This approach was intentionally considered to primarily allow for an inference about the respective changes of the GFB’s thermal and mechanical characteristics for the most critical stages of its operation.

### 4.1. Temporal Courses for the GFB’s Operational Characteristics

Figure 10 presents the temporal plots for the quantities that characterize the bearing’s operation identified during the experiments, including: speed of rotation, friction torque, temperature, and strain readings on the top foil.

As seen in Figure 10, the characteristic non-monotonic changes of the operational parameters accompany the air film development and its losing processes. Certain time periods are required for these processes to take place during both: (a) complete formulation of the thin air layer above the shaft’s journal, and (b) the final return to a dry friction contact, as the run-out stage proceeds and the shaft stops, eventually.

In Section 4.2, specific cases are declared by the authors with reference to the results presented in Figure 10, to allow for a more detailed analysis of the bearing’s behavior in Section 5. It should be noted that, although the experiments were conducted for the run-in top foil, a progressive process of its mechanical adjustment is still present in the bearing that continuously affects the measured strain and torque. In fact, no externally induced changes of the measured strains are seen within the last part of the stage of stable operation. Moreover, the stick-slip phenomenon was observed at various time moments, including the stage of cooling down, i.e., when the GFB does not operate. Specifically, there was a significant thermal shrinking appearing at no air film a dry friction contact is brought back during the run-out stage and then maintained while there is a break of the shaft. This results in both sudden and gradual changes in the measured friction torque. The greatest shift regarding the above-mentioned quantity is registered within the approximate time interval [820,835] seconds. Effectively, even though the value of the friction torque returns to zero immediately after the shaft stops, it still undergoes a considerable change later. This phenomenon, which is identified for a non-rotating shaft, is, however, considered to be out of the scope of the present study. Nonetheless, it does not significantly interfere with the general trends of the temperature and strain plots for the stage of cooling down. For the presented strain courses, an offset removal procedure was performed only once, at the measurement initialization, to visualize the total change of the identified strain gauges’ readings. As seen, a single run may cause small-amplitude elastic deformations of the top foil that are not completely canceled or, possibly, plastic ones may contribute.

### 4.2. Circumferential Distributions of Temperature and Strain in the Top Foil

**Figure 11:**
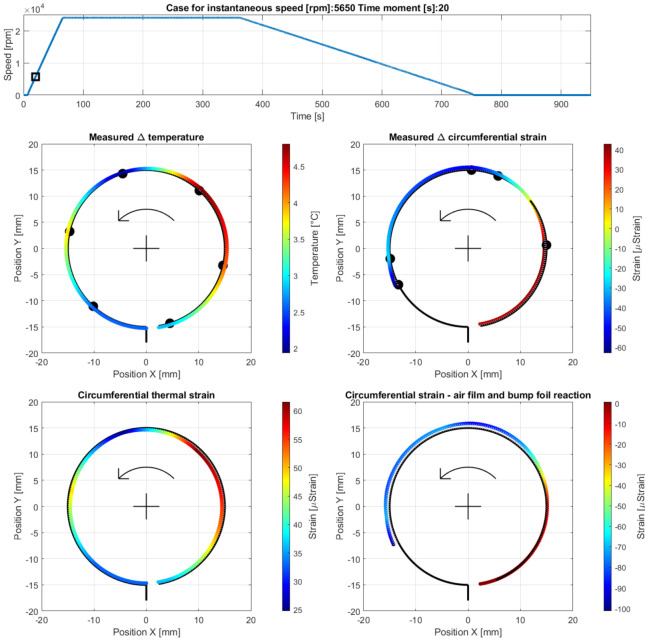
Cross-sectional views for the circumferential distribution of the temperature and strain identified in the top foil at 5650 rpm during the run-up stage of the GFB’s operation. The referential course of the rotational speed is provided on top. Its current value is visualized with a black square marked on the entire speed profile. The arrows indicate the direction of rotation of the shaft. The data presented in the cross-sectional views are explained in the current section.

Taking into consideration the registered temporal plots (Figure 10) and the overall assumptions regarding the investigated measurement scenario mentioned in Section 3.2, the following cases at various GFB’s operation stages were selected for a further, more comprehensive study on the thermomechanical properties of the tested bearing:

- Case A—phase of development of an air film during the run-up stage of the bearing’s operation at the measurement time moment 20 s, i.e., 13 s after the run-up stage was initiated (at 5650 rpm), specifically at the moment when the rates of the measured temperatures began to decrease. From that moment, the measured temperatures did not grow faster and faster anymore, until they finally reached the maximum level during the run-up stage of approximately 50 °C at 15,900 rpm. The results obtained for Case A are presented in Figure 11.

For the sake of clarity, having introduced Figure 11 as an example, the meaning of all cross-sectional views in the presented plots, as well as the assumed convention regarding the presentation of the signs of the visualized quantities is explained briefly here. The full color scale (i.e., from dark blue to dark red) is used to represent all the scatter of the visualized quantities, irrespective of their absolute values.

First, the two upper plots of the set of four circumferential views seen in Figure 11, respectively, present the temperature (on the left) and strain (on the right) distributions in the top foil. These plots make use of the equivalent (redundant) color mapping and shape scaling to visualize the values of the considered quantity. The exact measured values of these quantities are represented with black dots at given circumferential positions—following the prototype’s description found in Section 3. The spatial courses for both the temperature and strain along the curved top foil were found using linear interpolation and extrapolation. This approximation technique was also considered outside the angular range corresponding to the localizations of the integrated sensors to provide rough estimates of the mentioned quantities at the top foil-free end, as well as in the area where this foil is clamped to the bushing. However, the estimate for the strain at the top foil tie (i.e., outside the region covered by the strain gauges close to the foil’s tie) is not identified due to the expected, by the authors, complicated nature of the phenomena of the mechanical load carrying in this area. In fact, a total mechanical interaction between the bushing and the shaft’s journal includes force reactions that are completed via the foil’s tie. The lack of sensors in the mentioned region makes it impossible to reliably conclude a strain distribution in that place. 

As explained in Section 3, the strain gauges are mounted on the outer surface of the top foil. Hence, the positive values of the measured strain (specifically, the positive change of the strain found after initial offset level removal) indicate proceeding with a clamping of the foil around the shaft’s journal. In reference, the behavior of the top foil is represented by the reduced radius of the colored curve visualizing the strain distribution. Consequently, the negative strains are represented with the opposite change of the plotted curve’s radius. The positive strains generated due to thermal expansion (visualized in the bottom left plot in Figure 11) are modeled in a consistent way to provide the same interpretation of the behavior of the top foil—actually wrapping the shaft during the foil’s elongation in a continuous presence of the supporting bump foils and the caused contact limits. The thermal expansion coefficient applied to find the thermally induced strain in the top foil equals 12.8 × 10^−6^.

The results shown in the bottom right plot in Figure 11 visualize the circumferential strain approximating the hydrodynamic action of the air film complemented with a presumable contribution of the bump foils’ interaction. The presented strains were found by subtracting the calculated thermally induced strains (bottom left curve in Figure 11) from the resultant strains measured with the strain gauges (upper right curve in Figure 11). However, it should be noted that an additional assumption was made regarding the value of the strain visualized in the bottom right plot in Figure 11. Specifically, its value at the free end of the top foil was arbitrarily fixed to zero, which indicates the assumed realistic lack of a non-thermally induced contribution to the deformation of the mentioned part of the foil, irrespective of its actual displacement. Consequently, an adequate value of the strain was considered in the upper right curve in Figure 11 to proceed with the approximation procedure, of the magnitude equal to the thermally induced strain estimated for the mentioned localization and shown in the bottom left curve in Figure 1. The above-described correction was made to follow the assumption, considered by the authors, that only strains originating from the thermal expansion are found at the free end of the foil.

**Figure 12:**
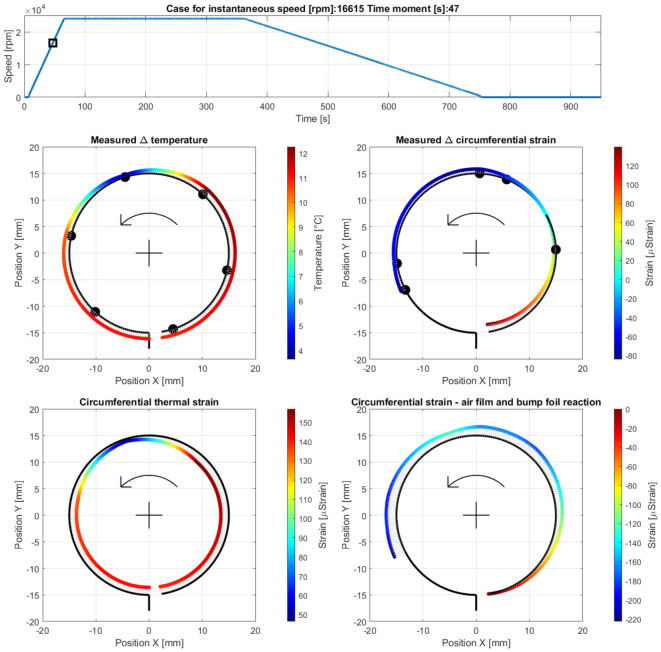
Cross-sectional views for the circumferential distribution of the temperature and strain identified in the top foil at 16,615 rpm during the run-up stage of the GFB’s operation before the homogenization of the temperature. The black square and arrows respectively indicate the actual rotational speed of the shaft and its direction.

**Figure 13:**
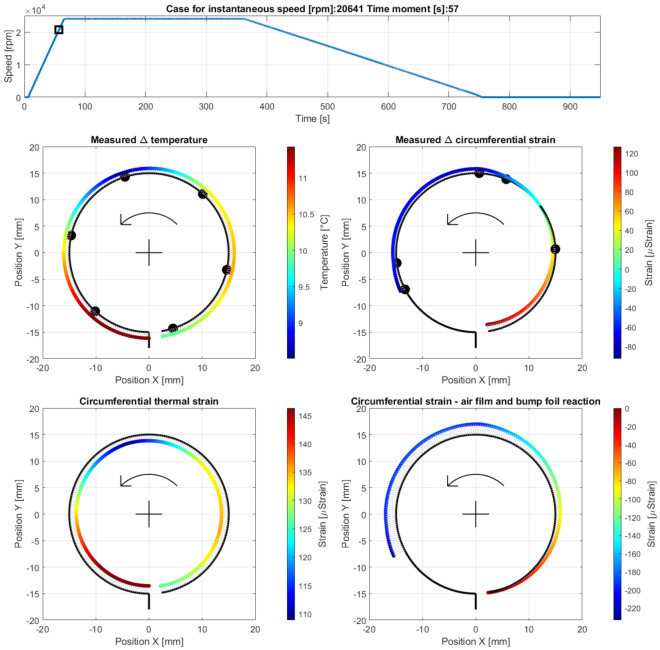
Cross-sectional views for the circumferential distribution of the temperature and strain identified in the top foil at 20,641 rpm during the run-up stage of the GFB’s operation after the homogenization of the temperature.

**Figure 14:**
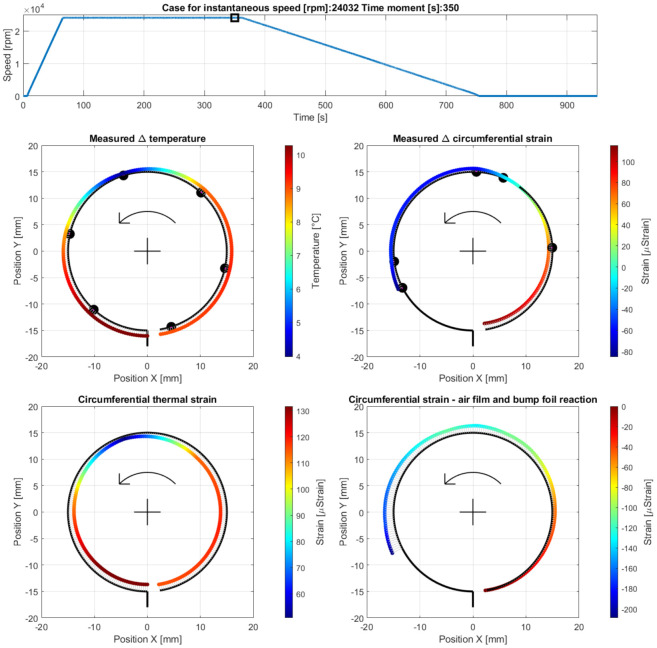
Cross-sectional views for the circumferential distribution of the temperature and strain identified in the top foil at 24,032 rpm during the stage of stable operation of the GFB.

**Figure 15:**
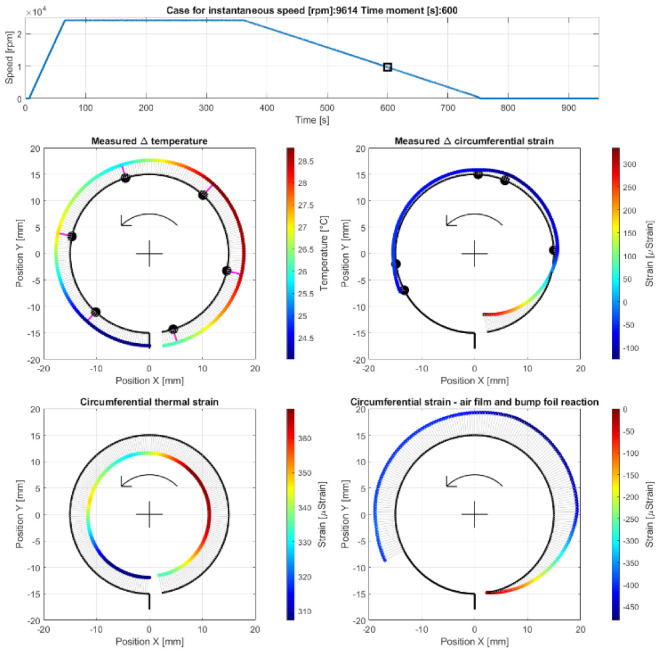
Cross-sectional views for the circumferential distribution of the temperature and strain identified in the top foil at 9614 rpm during the run-out stage of the GFB’s operation before the air film was completely lost.

**Figure 16:**
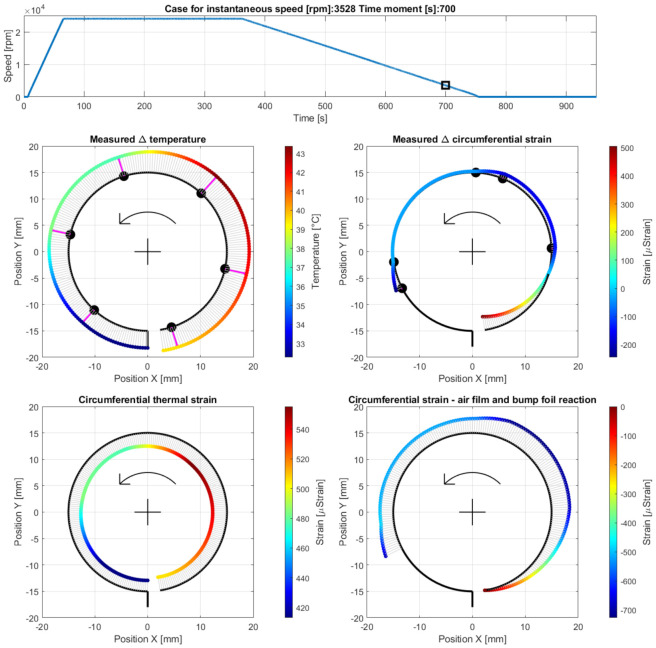
Cross-sectional views for the circumferential distribution of the temperature and strain identified in the top foil at 3528 rpm during the end of the run-out stage of the GFB’s operation after the air film was completely lost. The curves used to visualize the strains are rescaled by the factor 0.5 with respect to the previously shown cases.

**Figure 17:**
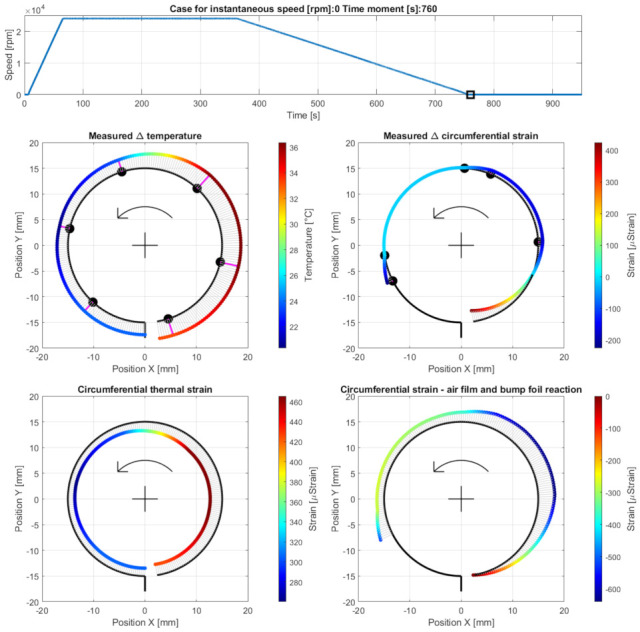
Cross-sectional views for the circumferential distribution of temperature and strain identified in the top foil at the stopped shaft. The curves used to visualize the strains are rescaled by the factor 0.5 compared to Cases A to F.

**Figure 18:**
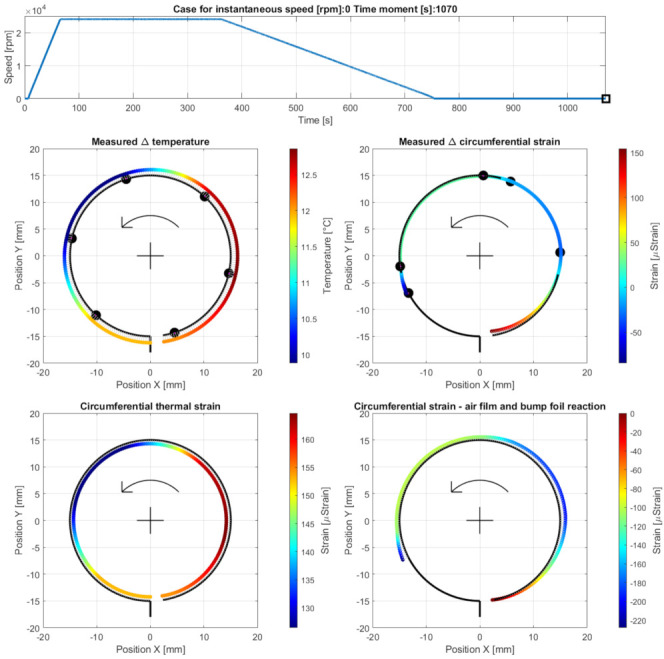
Cross-sectional views for the circumferential distribution of the temperature and strain identified in the top foil at the stopped shaft at the end of the measurements. The curves used to visualize the strains are rescaled by the factor 0.5 compared to Cases A to F.

- Case B—operation with a fully developed air film during the run-up stage, at the measurement time moment 47 s, i.e., 40 s after the run-up stage was initiated (at 16,615 rpm) before the temperature along the top foil became homogenized; Figure 12 presents the results obtained for the current case.

- Case C—operation with a fully developed air film during the run-up stage, at the measurement time moment 57 s, i.e., 50 s after the run-up stage was initiated (at 20,641 rpm) after the temperature in the top foil became homogenized (Figure 13).

- Case D—operation at the end of the stage of stable operation, at the measurement time moment 350 s, i.e., 283 s after the run-up stage was initiated (at 24,032 rpm); The results are presented in Figure 14.

- Case E—operation at the run-out stage before a complete loss of the air film, at the measurement time moment 600 s, i.e., 193 s after the run-out stage was initiated (at 9614 rpm); The results are presented in Figure 15.

- Case F—operation at the end of the run-out stage after a complete loss of the air film, at the measurement time moment 700 s, i.e., 293 s after the run-out stage was initiated (at 3528 rpm). The results are presented in Figure 16.

- Case G—initiation of the cooling down stage after the shaft’s stop, at the measurement time moment 760 s. The results are presented in Figure 17.

- Case H—end of the measurements during the cooling down stage at the time moment 1070 s. The results are presented in Figure 18.

**Figure 19:**
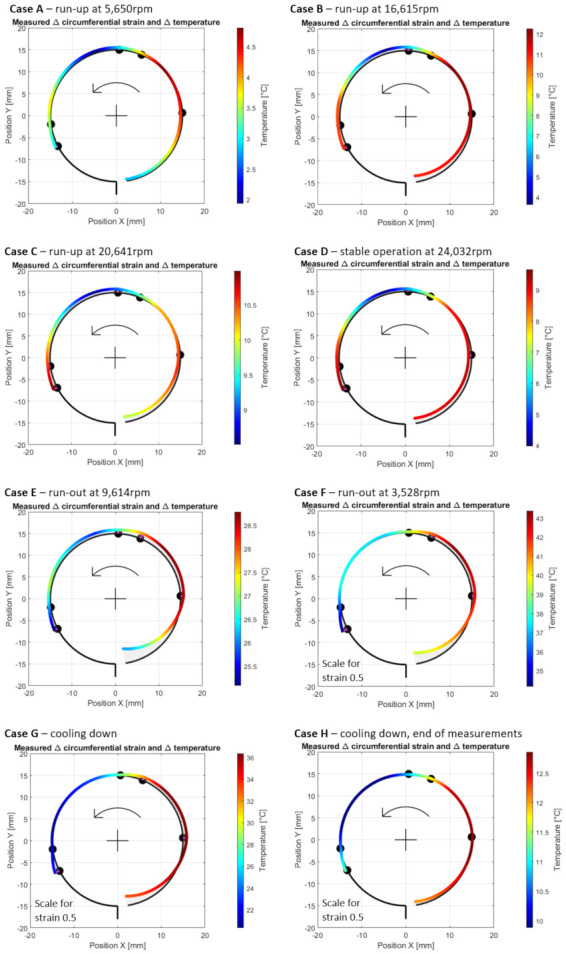
Compacted cross-sectional views for the circumferential distribution of the temperature and strain identified in the top foil for the studied cases (**A**–**H**), referencing the various stages of the GFB’s operation.

The aggregated data related to the circumferential distribution of the measured temperature and strain for all the above-introduced cases are shown in Figure 19. 

As previously mentioned, an analysis of the experimental outcomes is performed in Section 5.

## 5. Discussion

The following provides a discussion of the experimental outcomes obtained for the cases introduced in Section 4. These cases address, in the authors’ opinion, the most interesting phenomena appearing in the tested bearing during the subsequent stages of its operation.

### 5.1. Cases A–C—Development of an Air Film during Run-Up Stage

After the bearing’s shaft starts rotating, a sudden growth of the friction torque was identified (shown in Figure 10). This initial behavior of the GFB is natural, as it results from the dry friction present between the surfaces of the components which are in mechanical contact, i.e., the rotating shaft’s journal and the top foil. It should be highlighted that the presence of the protective layers on both cooperating elements prevented damage to the bearing. Otherwise, bearing malfunction inevitably occurs before the air film development is complete. Similarly, a positive rate of the temperature readings confirmed a not yet successfully finished process of the gaseous film generation. However, starting from the approximate speed of 5 krpm (Case A) the temperature gradually gets stabilized until it reaches its maximum value at approximately 15 krpm (Case B). Further increase of the rotational speed does not lead to any significant change of all of the measured parameters (Case C) that confirm the stable state of the previously generated air film.

It is worth mentioning that during the entire process of the air film development (Cases A–C) sudden and chaotic-looking changes of the strain registered by all strain gauges were observed. The continuous adjustment of the bump and top foils occurred in response to both film generation (via hydrodynamic effect) and variation of the friction torque (due to dry friction between the metallic parts). Moreover, the top foil underwent the phenomenon of a micro slip over the bump foils due to the progressive thermal expansion.

Before the gaseous film entirely develops (as addressed in case A), the highest temperatures, which are seen in Figure 11, were recorded in the upper right localization in the top foil, where it keeps contact with the rotating shaft. In fact, following the counter-clockwise direction of the rotation of the shaft, the GFB’s bushing tried to lift slightly and move to the left. The registered increase of the temperature along the GFB’s circumference, found with the applied approximation procedure, varied within the range from 2 °C up to 4.7 °C. The resulting thermal strain (in the order of 25–60 microstrains) amplified the effect of wrapping the top foil around the shaft.

The strain measured for Case A indicated the resultant wrapping effect at the free end of the top foil. As the speed increased (Case B), which meant simultaneous completion of the generation of the air film and further temperature change, the wrapping effect grew, which confirmed the hydrodynamic action. The maximum temperature was found at the top right zero-strain localization on the cross-sectional view in Figure 12. The dominant foil flattening effect was visible at its tie region. After a stable air film was generated (Case C), the temperature became homogenized along the circumference, even though it still reached relatively high values in the order of 11.5 °C above its ambient counterpart with the scatter equal to 3 °C. Moreover, for Case C, the maximum temperature was no longer found in the region where the air film was the thinnest. Contrarily, as shown in Figure 13, the raised temperature was observed within the region that squeezed the air before it entered the neck of the gaseous film. Accordingly, a sudden temperature drop occurred where the air expanded on top of the circumferential view. Next, i.e., moving counterclockwise, the air again became heated on the bottom—near the top foil tie.

### 5.2. Case D—Continuous Operation of the Bearing with a Stable Air Film

As seen in Figure 10, the values of all measured quantities remain unchanged. Only a slightly rising trend regarding the measured strains, which is found equal and common for all sensors, was observed. The GFB’s behavior did not change significantly for the constant rotational speed and non-changing external load. Adequately, no further deformation of the top foil was identified for the considered stage of operation. The amplitude of the friction torque was approximately ten times smaller than its counterpart, measured during both the run-up and run-out stages of the GFB’s operation.

The above-discussed effect of squeezing and expanding the air film around its neck is visible in Figure 14. Similar local changes in the temperature distribution were found. Moreover, again, no resultant in-plane strain was identified around the neck of the gaseous film which confirmed the expected presence of radial forces (originated from the hydrodynamic effect) as dominant ones for the considered angular localization.

### 5.3. Cases E and F—Run-Out Stage and Gradual Loss of the Air Film

First, during the initial phase of the run-out stage of operation (i.e., during Case E and earlier), continuous growth of the friction torque and temperature was observed. In fact, since the rotational speed decreased, the air film underwent gradual destruction. The occurrence of a dry friction in the area where the bushing and shaft cooperate led to a sudden growth of both heat generation and temperature, as visualized in Figure 15. These symptoms clearly indicate a change in the bearing’s operational parameters, specifically regarding the state of the air film. Finally, when the rotational speed became insufficient to continuously maintain the gaseous part of the supporting layer, a dry friction contact appeared and remained until the shaft stopped (Case F).

Low deceleration of the rotational speed resulted in a relatively high temperature of the top foil, from 33 °C up to 43 °C above the ambient temperature, which caused significant thermal expansion. The highest temperatures for Cases E and F were found in the dry friction contact region. The analysis of the strains led to observations similar to those found for Cases A–C. Specifically, the top foil tended to wrap around the shaft at its free end and, contrarily, got flattened on top and on the left of the cross-sectional data presentation, as shown in Figure 16. The amplitudes of strains, however, were approximately four times greater during the run-out stage.

### 5.4. Cases G and H—Cooling Down

The strains registered for Case G did not converge to their initial values, i.e., at the moment of measurement initialization. The large scatter of the strains, which are shown in Figure 17 and were observed at the beginning of the cooling down stage, resulted from both high temperatures (i.e., due to thermal expansion, which is a reversible phenomenon) and the progressive process of the foil’s shape adjustment. A single run of the bearing led to the irreversible change of the locations of all foils, and, possibly, to the plastic deformation of the top foil. These phenomena naturally occurred during the bearing’s operation due to the process of losing the air film, progressive thermal expansion and, finally, resulted from the force response of the bump foils.

The identified gradual temperature drops followed the expected change of the generated heat energy. In fact, when the shaft was stopped, no source of heat originating from the evolving friction conditions was present in the bearing any longer. At the end of measurements (which is addressed by Case H), the registered temperature scatters decreased to achieve the interval from 10 °C up to 13 °C above the ambient temperature (Figure 18).

## 6. Summary and Concluding Remarks

The conducted research experimentally confirmed the demanded measurement functionality for the designed and constructed specialized sensing top foil that was successfully installed in a normally operating GFB prototype. Specifically, the novel approach and the newly proposed technical solution, which makes use of the integrated thermocouples and bonded strain gauges, allowed for the acquisition of the temperature and strain readings at selected localizations in the outer surface of the top foil.

The present study is considered by the authors as an attempt of a comprehensive characterization of the bearing which may help to identify both the causes of the top foil’s deformations, i.e., the change of the top foils’ geometry and the thermomechanical conditions at which an air film develops due to the hydrodynamic action. The conducted, primarily qualitative, investigation deals with the two interacting physical domains, having considered circumferential distribution of the temperature and strain.

With the spatial courses of the measured quantities, the critical regions that contribute to the phenomena important for the GFB’s operation may be clearly indicated. Specifically, the angular position of the region exhibiting the raised temperature during both the run-up and run-out stages showed the part of the top foil’s surface where a dry friction contact was present. Moreover, a characteristic local decrease of the temperature on top of the rotating shaft found during the stage of a stable operation (as shown in Figure 14) indicated the large flow of the air being expanded when coming out of the area of the thinnest gaseous film. The adequate greater curvature of the top foil (i.e., local wrapping of the shaft), which is seen on the right, and its simultaneous flattening on the left with respect to the above-mentioned region confirmed the resultant shift of that foil due to the hydrodynamic interaction.

The authors are aware of the existing limitations of the present study. First, the temperature and strain readings were acquired and analyzed only for the central cross-section of the tested bearing. Moreover, the floating configuration without the external load was investigated, which stood for the first step of the analysis—prior to the planned use of a two-node support configuration of a GFB [43]. Next, significant geometric modifications of the foils’ geometry, with the intentionally introduced gaps (incisions) in the bump foils, as well as the measurement method used to identify temperature and strain, made the proposed technical solution not yet ready to be implemented in commercial cases. The obtained preliminary results, however, allow for the inspection of the relation between the mechanical and thermal properties of the tested bearing while it runs at all subsequent stages of operation.

There are also several issues that need to be addressed during further studies. First, the existing technical limitations related to the strain measurements at the top foil’s free end and its tie make it difficult to reliably estimate the foil’s behavior (deformation) at these localizations. Hence, other measurement techniques should be possibly proposed and then used to fill in the existing gap and complete the circumferential distribution of the strain in the top foil. Next, the installed strain gauges caused local changes in the top foil’s stiffness (overstiffening) which may influence the process of developing the air film and proper bearing’s operation in the case of other configurations of GFBs, characterizing various shaft’s diameters, bearing’s widths, loads, and rotational speeds. The authors admit that each modification of the bearing should be subjected to an additional study to reliably formulate general conclusions on a given generation of GFBs. In the authors’ opinion, however, the proof of concept for the investigated measurement technique was successful. Moreover, the repeatability of the temperature and strain measurements should be assessed. Finally, even though the strains were measured accurately, it is not a trivial task to decide when their offset values should be cancelled and, in turn, when the tracking of the temporal courses for the strains should begin. In fact, the respective temporal force (pressure) contributions of both the air film and the bump foil to the measured resultant strains in the top foil are not yet experimentally investigated.

## Figures and Tables

**Table 1:** Geometric characteristics of the tested bearing.

Parameter/Characteristics	Value/Description
Shaft’s journal nominal diameter	30 mm
Bushing’s inner nominal diameter	31 mm
Bearing width	40 mm
Foil thickness	0.1 mm
Characteristics of the bump foils:	
Bump pitch	4.8 mm
Bump height	0.4 mm
Bump length	3.4 mm
Number of bumps in segment	7
Number of segments	4
Number of foils	3
Theoretical nominal clearance	0.02 mm

## Data Availability

The data presented in this study are available on request from the corresponding author.
